# A Multi-Model Machine Learning Framework for Identifying Raloxifene as a Novel RNA Polymerase Inhibitor from FDA-Approved Drugs

**DOI:** 10.3390/cimb47050315

**Published:** 2025-04-28

**Authors:** Nhung Thi Hong Van, Minh Tuan Nguyen

**Affiliations:** 1 Department of Physiology, Dongguk University College of Medicine, Gyeongju 38066, Republic of Korea; vthn0295@dgu.ac.kr; 2 College of Pharmacy, Dongguk University, Seoul 04620, Republic of Korea

**Keywords:** RNA-dependent RNA polymerase, machine learning, deep learning, raloxifene, antiviral

## Abstract

RNA-dependent RNA polymerase (RdRP) represents a critical target for antiviral drug development. We developed a multi-model machine learning framework combining five traditional algorithms (ExtraTreesClassifier, RandomForestClassifier, LGBMClassifier, BernoulliNB, and BaggingClassifier) with a CNN deep learning model to identify potential RdRP inhibitors among FDA-approved drugs. Using the PubChem dataset AID 588519, our ensemble models achieved the highest performance with accuracy, ROC-AUC, and F1 scores higher than 0.70, while the CNN model demonstrated complementary predictive value with a specificity of 0.77 on external validation. Molecular docking studies with the norovirus RdRP (PDB: 4NRT) identified raloxifene as a promising candidate, with a binding affinity (−8.8 kcal/mol) comparable to the positive control (−9.2 kcal/mol). The molecular dynamics simulation confirmed stable binding with RMSD values of 0.12–0.15 nm for the protein–ligand complex and consistent hydrogen bonding patterns. Our findings suggest that raloxifene may possess RdRP inhibitory activity, providing a foundation for its experimental validation as a potential broad-spectrum antiviral agent.

## 1. Introduction

RNA-dependent RNA polymerase (RdRP) represents a critical enzyme found across numerous RNA viruses that cause significant human diseases [1]. This essential enzyme serves as a fundamental model for understanding viral replication mechanisms and developing antiviral strategies. Through RdRP research, scientists have gained valuable insights into viral enzymes responsible for diseases like hepatitis C [2], norovirus infections [3], and COVID-19 [4], making it a cornerstone of antiviral research.

The enzyme’s mechanism is remarkably sophisticated, catalyzing viral RNA genome synthesis through specific template recognition and nucleotide incorporation. During both initiation and elongation phases, RdRP forms intricate molecular interactions with viral and cellular cofactors, a process that parallels across many disease-causing RNA viruses [5]. Understanding these interactions has proven crucial for developing treatments for various viral infections, as similar binding mechanisms are observed in viruses causing gastrointestinal, respiratory, neurological, and hepatic diseases.

The enzyme’s structural features, particularly its active site architecture and template-binding channel, are highly conserved across RNA virus families and determine the specificity and efficiency of RNA synthesis [6]. These characteristics make 3D polymerase (3Dpol), a specific type of RdRP found in picornaviruses [7] and noroviruses [8], an attractive target for antiviral drug screening, as disrupting its binding interactions can effectively inhibit viral replication.

Raloxifene functions as a selective estrogen receptor modulator (SERM), acting as both a partial activator and blocker of estrogen receptors [9]. It produces estrogen-like effects on bone tissue while blocking estrogen’s effects in breast and uterine tissues [9]. The medication received FDA approval in 1997 and is now available in generic form [9].

**Figure 1:**
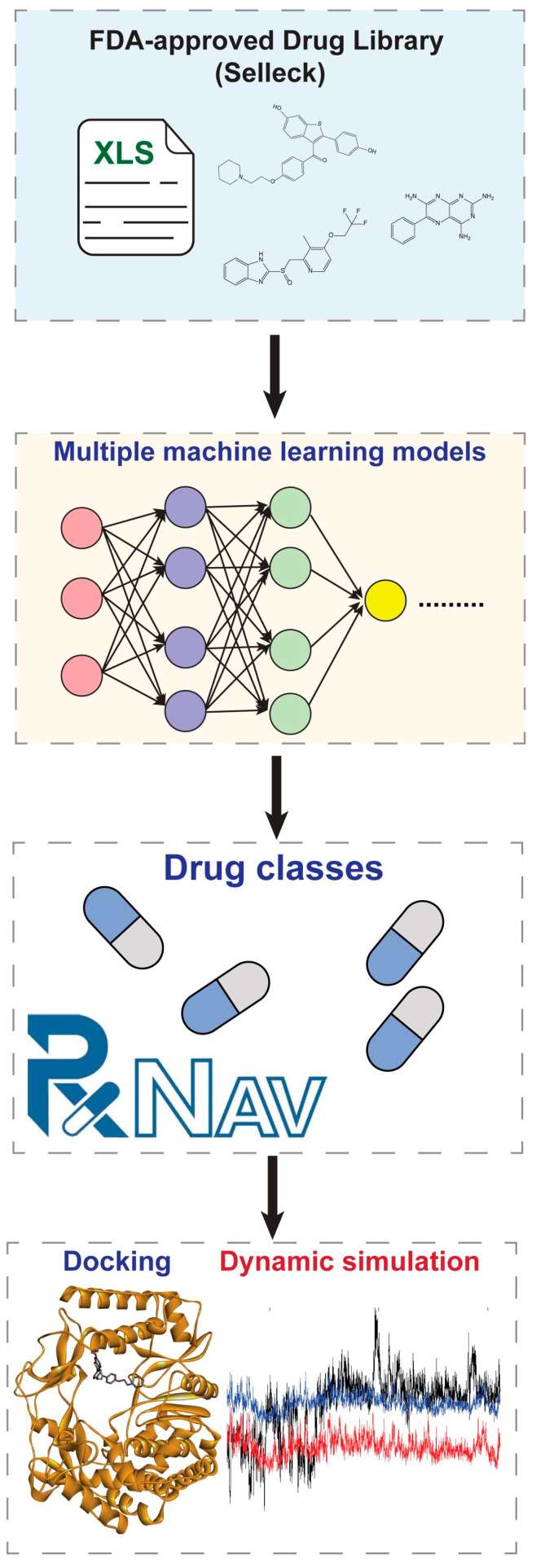
The workflow of the current study.

In this study, we developed a comprehensive computational framework for identifying potential viral RNA polymerase inhibitors. Our approach utilized a well-curated dataset from PubChem (AID 588519), comprising 152,580 compounds with known activities against viral RNA polymerase (3Dpol), including 1006 active and 151,574 inactive compounds [10,11]. This dataset served as the foundation for developing both traditional machine learning and Convolutional Neural Network (CNN) deep learning models. We implemented LazyClassifier to systematically evaluate multiple traditional machine learning algorithms, enabling the identification of the most effective classification models for activity prediction. These complementary machine learning approaches were then applied to screen an FDA-approved drug library and found raloxifene as a potential RdRP inhibitor. This computational pipeline integrates multiple prediction models to enhance the reliability of our virtual screening results, providing a robust foundation for identifying promising drug candidates for experimental validation. The workflow of this study is demonstrated in Figure 1.

## 2. Methods

### 2.1. Traditional Machine Learning

The dataset comprised 1006 active compounds and 1000 inactive compounds randomly selected from a pool of 151,574 inactive compounds (https://pubchem.ncbi.nlm.nih.gov/bioassay/588519, accessed on 20 October 2024) [10,11]. An external validation set was created by randomly selecting an additional 1000 inactive compounds from the 150,574 remaining inactive compounds. To enhance model performance, compounds with molecular weights below 200 or above 500 g/mol were excluded from the analysis.

The RDKit (version 2022.09.5) cheminformatics toolkit [12] was used to process Simplified Molecular Input Line Entry System (SMILES) strings and generate Morgan fingerprints (radius 2, 2048 bits) for each molecule. These structural fingerprints were converted to NumPy arrays, while activity labels were transformed into binary values (active = 1, inactive = 0). The curated dataset was then split into training (80%) and testing (20%) subsets using scikit-learn’s train_test_split package 1.6.1.

The LazyClassifier from the LazyPredict package v0.2.13 was employed to automatically train and evaluate multiple classification algorithms simultaneously [13]. The accuracy, ROC-AUC, and F1 score represented the percentage of all correct predictions, the model’s ability to distinguish between classes, and the harmonic mean of precision and recall. The five highest-performing models were identified based on training performance metrics and subsequently validated using both the testing subset and the external validation set.

### 2.2. Convolutional Neural Network (CNN) Deep Machine Learning

In our deep learning approach, we developed a CNN using the TensorFlow/Keras framework [14,15,16]. The network began with an input layer designed to process 2048-dimensional molecular fingerprints, which fed into two sequential convolutional layers. Each convolutional layer utilized 16 filters with a kernel size of 11 and featured batch normalization alongside ReLU activation functions to improve convergence and enhance feature detection capabilities. After the convolutional layers, we incorporated a max pooling layer with a pool size of 2 to reduce dimensionality while retaining essential features. The architecture then included a flattening operation followed by two fully connected dense layers with 128 and 64 units, respectively, both using ReLU activation. Dropout layers (rate = 0.3) were inserted between dense layers to mitigate overfitting. The output layer employed sigmoid activation for binary classification, producing probability scores for each molecular fingerprint. For model training, we implemented an Adam optimizer with a learning rate of 0.0001 and binary cross-entropy as the loss function. The model was trained for 500 epochs with a batch size of 25, utilizing early stopping (patience = 30, monitoring validation loss) to prevent overfitting. The curated dataset was then split into training (80%) and testing (20%) subsets using scikit-learn’s train_test_split package 1.6.1. A thousand inactive compounds were also used to examine the model. Model performance was evaluated using multiple metrics, including precision, recall, and specificity.

Traditional classifier models and the CNN deep learning model were built using the Python language (version 3.9.5) on Visual Studio Code version 1.99.3, with TensorFlow 2.10.0.

### 2.3. FDA-Approved Drug Library

The FDA-approved drug library was retrieved from the website of Selleck Chemical LLC (Houston, TX, USA, https://www.selleckchem.com, accessed on 25 April 2025). Compounds without a 3D structure were excluded. The remaining drugs were handled similarly to the dataset, then predicted by the models.

The dataset and models in this study are available in the GitHub repository: https://github.com/vthn0295/raloxifene-RdRp, accessed on 25 April 2025.

### 2.4. RxNav Drug Classification

To identify and classify available drugs in our compound library, we used the RxNorm RESTful API (RxNav) provided by the National Library of Medicine. A Python-based classifier was developed to systematically categorize 221 compounds based on their therapeutic and pharmacological properties. The classifier interfaced with the RxNav API (https://mor.nlm.nih.gov/RxClass/, accessed on 20 January 2025) to retrieve standardized drug classification information.

### 2.5. Molecular Docking

The RdRP protein structure (PDB ID: 4NRT [17]) was obtained from the Protein Data Bank. Compound 6, a Suramin derivative, was selected as a positive control because it was shown to bind specifically to a site located in the polymerase thumb domain of norovirus RNA-dependent RNA polymerase (NV-RdRP). The sulfonate groups on the naphthalene sulfonic head of compound 6 establish strong interactions with the RNA binding loop (residues 433–440). In addition, compound 6 significantly inhibited the activity of both human and murine norovirus RdRPs with IC50 values of 1000 ± 90 nM and 115 ± 15 nM, respectively. Therefore, compound 6 is an appropriate positive control to benchmark the binding interactions and potential inhibitory effects of candidate compounds. Prior to docking, crystal ligands and water molecules were removed from the structure. The protein was prepared using AutoDock Tools version 1.5.6, which included the addition of polar hydrogen atoms and Kollman charges, followed by conversion to pdbqt format. Ligand structures were initially designed in ChemDraw Ultra 12.0 [18] and subsequently processed using Open Babel 3.1.1 [19] to add hydrogen atoms and convert to pdbqt format.

Molecular docking simulations were performed using AutoDock Vina version 1.1.2. A cubic grid box with dimensions of 18.5 Å along all axes was used for docking calculations, following the modified protocol from our previous publication [20]. The resulting protein–ligand interactions were analyzed and visualized using Open-Source PyMOL [21].

### 2.6. Molecular Dynamics Simulation

The molecular dynamics simulation was conducted using GROMACS 2024.1 [22] with a slightly modified methodology from the previous study [23]. System preparation was accomplished through the CHARMM-GUI interface, with parameters assigned using the CHARMM36 force field. The protein–ligand complex was solvated in an explicit TIP3P water model, and the system was neutralized by adding potassium chloride ions. The simulation was performed under NPT conditions (constant number of particles, pressure, and temperature) at 300 K and 1 atm. Throughout the 30-nanosecond production run, trajectory and energy data were recorded at 10-picosecond intervals to analyze the dynamic behavior of the complex.

## 3. Results

### 3.1. Performance Evaluation of Machine Learning Classifiers for RNA Polymerase Inhibitor Prediction

**Figure 2:**
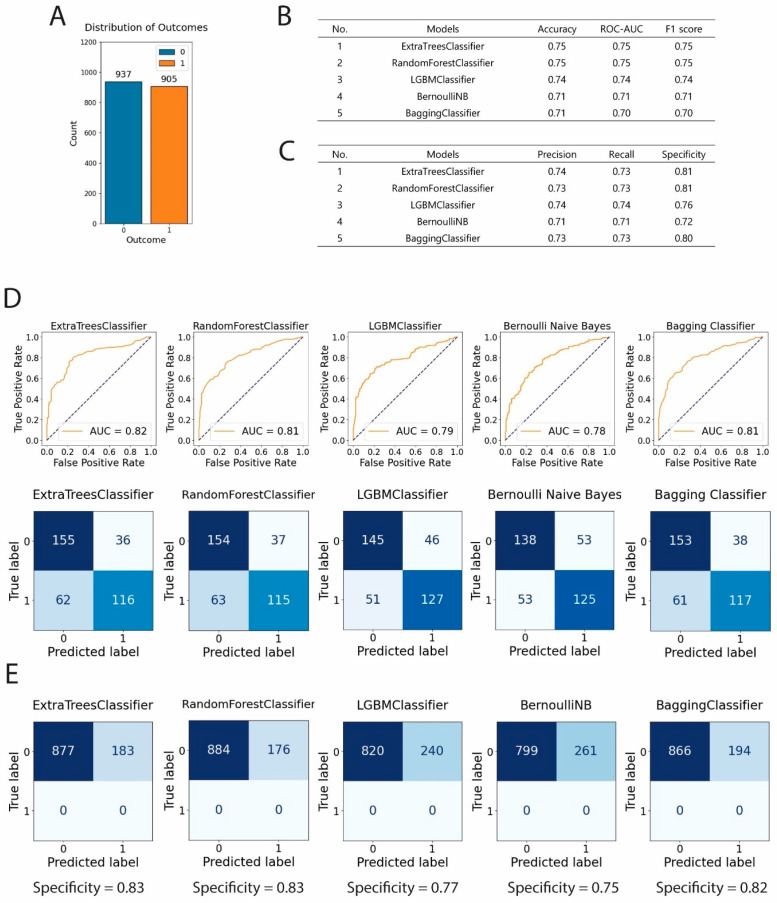
Evaluation of the top five machine learning classifiers. (**A**) Distribution of compounds in the dataset showing 937 inactive (0) and 905 active (1) compounds. (**B**,**C**) Performance metrics comparing ExtraTreesClassifier, RandomForestClassifier, LGBMClassifier, BernoulliNB, and BaggingClassifier across accuracy, ROC-AUC, F1 score, precision, recall, and specificity. (**D**) ROC curves and confusion matrices for each classifier showing detailed performance in distinguishing between active and inactive compounds (upper panel). Performance evaluation of machine learning models through confusion matrices using the validation dataset (lower panel). (**E**) Confusion matrix analysis of model performance testing using 1000 external inactive compound inputs. The tested models were from Figure 2B.

We first evaluated multiple machine learning classifiers to identify the most effective models for predicting RNA polymerase inhibitory activity. Our dataset distribution (Figure 2A) comprised a well-balanced collection of 937 inactive (0) and 905 active (1) compounds, providing a robust foundation for model development.

Through our systematic evaluation of classification algorithms, we identified five high-performing models (Figure 2B). The ExtraTreesClassifier and RandomForestClassifier demonstrated superior performance, with identical accuracy, ROC-AUC, and F1 scores of 0.75. The LGBMClassifier followed closely, with marginally lower metrics of 0.74 across all three evaluation criteria. The BernoulliNB and BaggingClassifier models achieved accuracy and F1 scores of 0.71, with ROC-AUC values of 0.71 and 0.70, respectively.

We conducted a detailed precision–recall analysis to further characterize model performance (Figure 2C). The ExtraTreesClassifier achieved a precision of 0.74 and recall of 0.73, while the RandomForestClassifier showed precision and recall of 0.73. The LGBMClassifier displayed balanced precision and recall values of 0.74, while the BaggingClassifier demonstrated precision and recall of 0.73. Regarding specificity, we observed that the ExtraTreesClassifier and RandomForestClassifier both attained the highest scores of 0.81, followed by the BaggingClassifier (0.80), LGBMClassifier (0.76), and BernoulliNB (0.72).

To visualize discriminative performance, we generated ROC curves for each classifier (Figure 2D, upper panel), with AUC values ranging from 0.78 to 0.82, confirming their strong predictive capabilities. The confusion matrices further validated model effectiveness (Figure 2D, lower panel), illustrating the distribution of true positives, true negatives, false positives, and false negatives across all five models.

We subsequently tested our models with an external validation set comprising 1000 independent inactive compounds (Figure 2E). All five classifiers maintained robust specificity, with the ExtraTreesClassifier and RandomForestClassifier both achieving specificity of 0.83, followed by the BaggingClassifier (0.82), LGBMClassifier (0.77), and BernoulliNB (0.75). These results confirmed the generalizability of our models to unseen data and their suitability for subsequent virtual screening applications.

### 3.2. Evaluation and Performance Analysis of Convolutional Neural Network Deep Learning Model for RNA Polymerase Inhibitor Prediction

**Figure 3:**
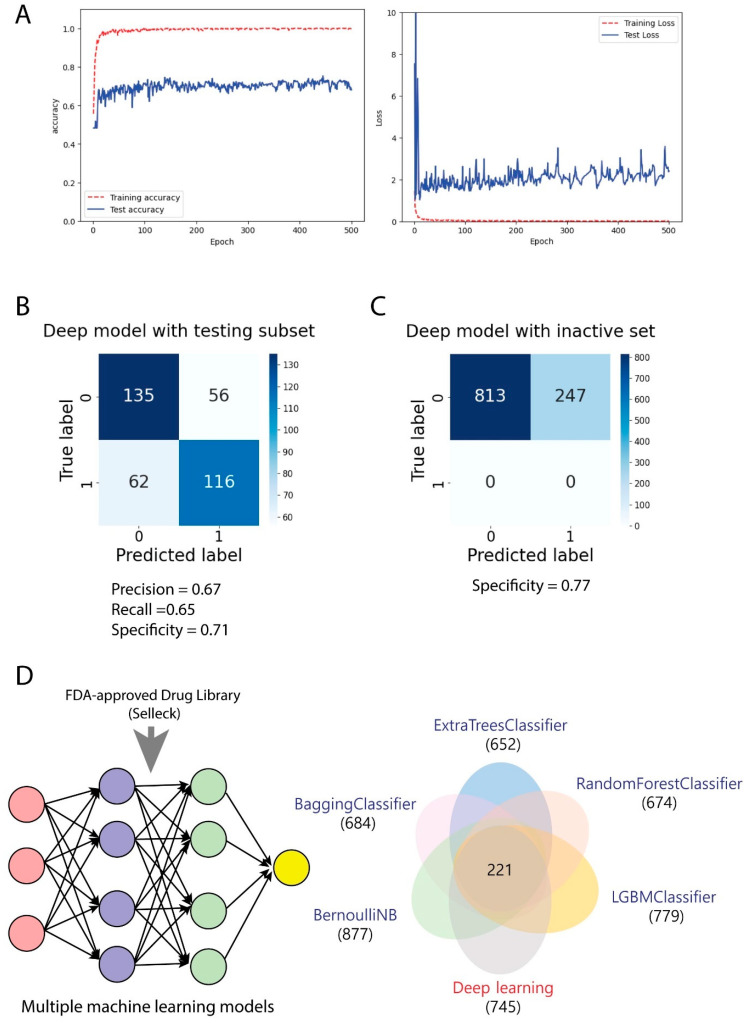
Evaluation of convolutional neural network (CNN) deep learning model. (**A**) Learning curves over 500 epochs showing the model’s training progression. (**B**) Confusion matrix for deep learning model performance on validation data. (**C**) Confusion matrix analysis for deep learning model performance using 1000 external inactive compounds inputs. The tested models were from Figure 3B. (**D**) Schematic representation of the multi-model machine learning framework for screening the FDA-approved drug library (Selleck) (Left panel). Venn diagram showing the distribution of predicted compounds across five different classifiers from Figure 2B and CNN deep learning model from Figure 3A, with 221 common compounds correctly identified by all six models (Right panel).

We conducted a comprehensive evaluation of our convolutional neural network (CNN) deep learning model to assess its effectiveness in predicting RNA polymerase inhibitory activity. As illustrated in Figure 3A, the learning curves over 500 epochs demonstrated consistent improvement in model performance, with training accuracy (red line) reaching nearly 1.0 and test accuracy (blue line) stabilizing around 0.7, indicating successful training while avoiding overfitting.

Our validation of the CNN deep learning model through confusion matrix analysis (Figure 3B) revealed a precision of 0.67, recall of 0.65, and specificity of 0.71. The model correctly classified 116 active compounds and 135 inactive compounds, with 56 false positives and 62 false negatives. While these metrics were slightly lower than those of traditional machine learning approaches, they provided complementary predictive value to our overall framework.

When challenged with the external validation set of 1000 inactive compounds (Figure 3C), our CNN model maintained a strong specificity of 0.77, correctly identifying 813 inactive compounds while generating 247 false positives. This performance demonstrated the model’s ability to generalize to unseen data and its utility as a screening tool for identifying potential RNA polymerase inhibitors.

We provided a comprehensive visualization of our multi-model machine learning framework’s application to FDA-approved drugs (Figure 3D). The left panel illustrates the systematic workflow for screening the FDA-approved drug library from Selleck Chemical LLC using our six machine learning models (five traditional classifiers and the CNN deep learning model). This approach allowed us to process and evaluate diverse drug candidates across multiple therapeutic categories for potential RNA polymerase inhibition activity. The right panel features a Venn diagram that quantifies the distribution of predicted compounds across all classifiers when applied to the FDA-approved drug library. This analysis revealed that 221 FDA-approved compounds were consistently identified as potential RNA polymerase inhibitors by all six models, highlighting a high-confidence consensus set. Additionally, the diagram demonstrates the unique predictive contributions of each model, with LGBMClassifier identifying 779 unique compounds, followed by BernoulliNB (877), BaggingClassifier (684), RandomForestClassifier (674), ExtraTreesClassifier (652), and CNN deep learning model (745). This multi-model approach significantly enhanced the reliability of our screening results by leveraging the complementary strengths of diverse algorithms in identifying repurposable FDA-approved drugs as novel RNA polymerase inhibitors.

### 3.3. Drug-Target Network Analysis and Molecular Docking Results

**Figure 4:**
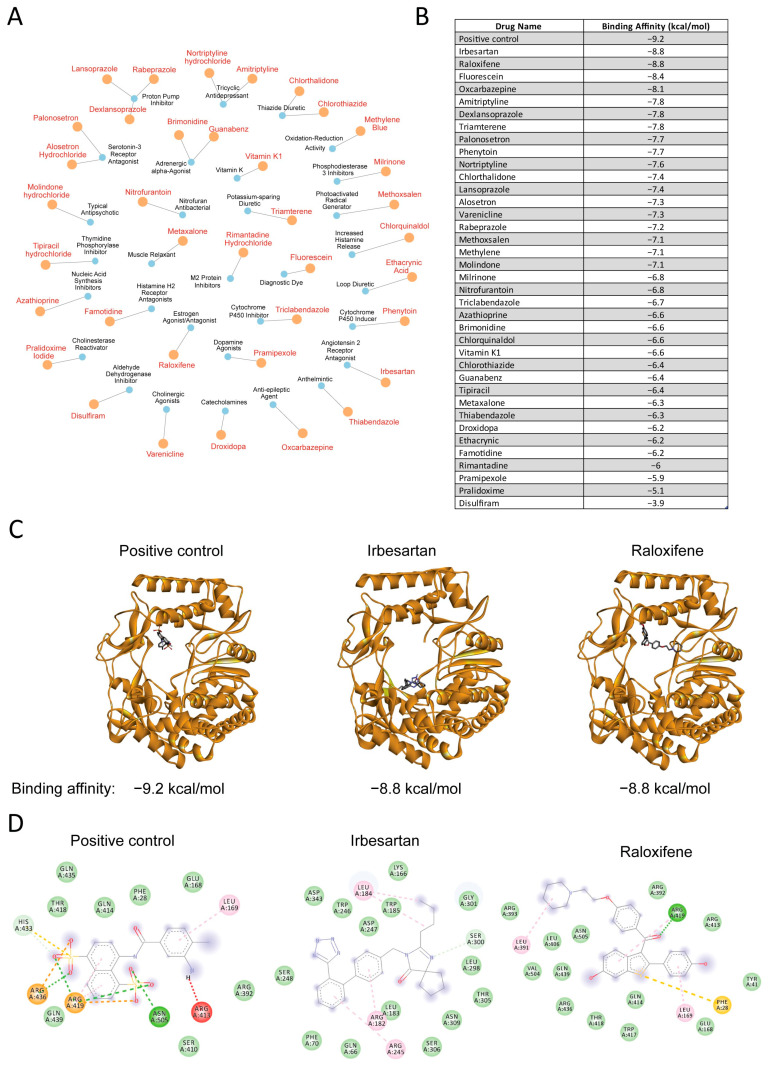
Drug-target network and molecular docking studies of repurposed drug candidates. (**A**) Network visualization displaying drugs (orange nodes) and their therapeutic targets or mechanisms of action (blue nodes). (**B**) Ranked list of drugs and their corresponding binding affinities (kcal/mol). (**C**) Three-dimensional molecular docking poses of positive control, irbesartan, and raloxifene with the RNA-dependent RNA polymerase (RdRP) (PDB ID: 4NRT). (**D**) Two-dimensional interaction diagrams showing key protein–ligand interactions based on the molecular docking models from Figure 4C.

Next, we employed the RxNav tool from the National Library of Medicine to conduct a comprehensive analysis and secondary filtration of our 221 repurposed drug candidates from Figure 3D. This systematic approach ensured we focused exclusively on drugs with current regulatory approval and well-established safety profiles. To visualize the complex therapeutic relationships and mechanism diversity within this filtered dataset, we constructed a detailed drug-target network (Figure 4A). This network mapped FDA-approved drugs (represented as orange nodes) and their corresponding therapeutic mechanisms or targets (represented as blue nodes), enabling us to characterize the diverse pharmacological landscape of our identified drugs.

We selected the thumb site inhibitor complex (PDB ID: 4NRT) as our molecular docking model, based on our observation that the catalytic active site within the thumb subdomain is highly conserved across RNA virus families. Through systematic molecular docking studies, we generated a ranked list of potential thumb site RNA polymerase inhibitors (Figure 4B). Our analysis revealed binding affinities spanning from −9.2 to −3.9 kcal/mol. We found that raloxifene emerged as a particularly promising candidate, demonstrating a binding affinity of −8.8 kcal/mol that closely matched our positive control (−9.2 kcal/mol). While we observed that irbesartan exhibited comparable binding energy (−8.8 kcal/mol), we noted its binding pose deviated significantly from the positive control.

To better understand the binding modes, we performed three-dimensional molecular docking analysis (Figure 4C). Our investigation revealed striking similarities between raloxifene’s binding pose and the positive control within the RdRP binding pocket. We observed that both compounds adopted similar spatial positions and orientations within the binding site, leading us to hypothesize that raloxifene might inhibit RdRP through mechanisms analogous to known inhibitors. In contrast, despite irbesartan’s strong binding affinity, we found its spatial orientation diverged notably from the positive control.

To validate these findings, we analyzed detailed two-dimensional interaction diagrams (Figure 4D). Our examination provided additional support for raloxifene’s potential as an RdRP inhibitor, as we identified key protein–ligand interactions that paralleled those of the positive control, including matching patterns of hydrogen bonds and hydrophobic interactions.

Based on our comprehensive analyses, we identified raloxifene as a promising candidate for further investigation as an RdRP inhibitor, supported by both its structural similarities to the positive control and favorable binding energy profile.

### 3.4. Molecular Dynamics Simulation Analysis Reveals Stable Binding Between RdRP and Raloxifene

**Figure 5:**
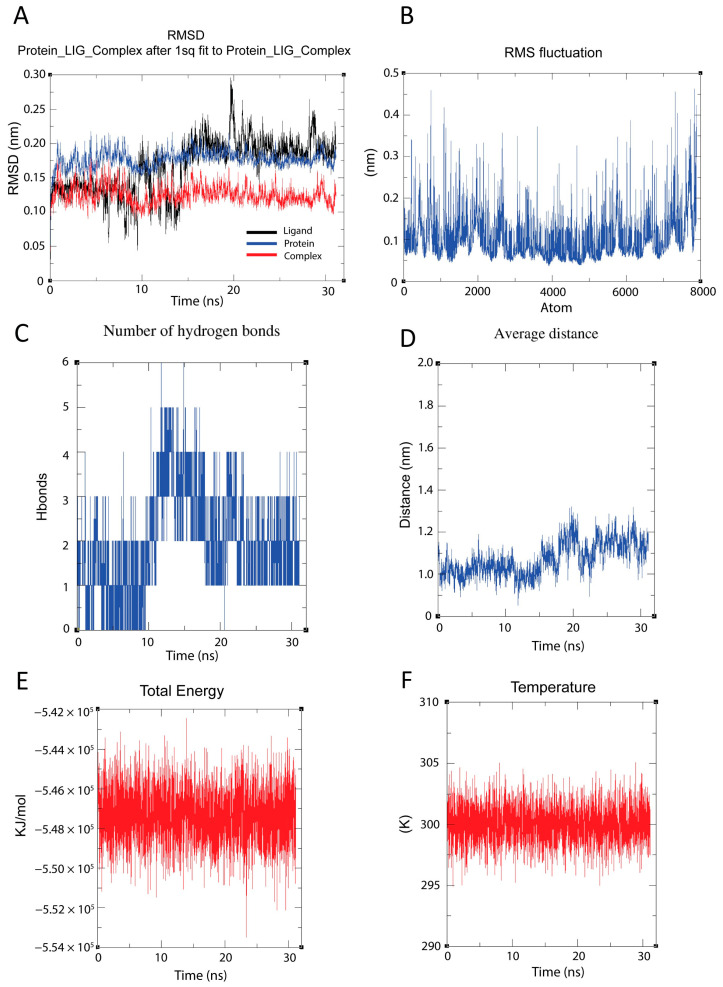
Molecular dynamics simulation analysis of RdRP (protein)–raloxifene (ligand) complex stability and interactions over 30 nanoseconds (ns). (**A**) Root mean square deviation (RMSD) analysis demonstrating the conformational evolution of the ligand (black), protein backbone (blue), and complete protein–ligand complex (red). Values were calculated after least-squares fitting to the initial structure. (**B**) Root mean square fluctuation (RMSF) per atom, calculated over 8000 atoms. Values represent atomic positional fluctuations from their time-averaged positions. (**C**) Time-dependent analysis of intermolecular hydrogen bonds between the protein and ligand, measured as the total number of hydrogen bonds present at each time point. (**D**) Average distance evolution between protein and ligand centers of mass throughout the simulation, measured in nanometers (nm). (**E**) Total energy profile of the system, including potential and kinetic energy contributions, measured in kJ/mol. (**F**) Temperature regulation profile maintaining the system at 300 K. Temperature is measured in Kelvin (K).

We conducted a 30-nanosecond molecular dynamics simulation to evaluate the stability and dynamic interactions of the RdRP–raloxifene complex. To assess conformational stability, we first analyzed the RMSD patterns (Figure 5A), which demonstrated stable conformational behavior across all components. We observed that the protein backbone (blue) maintained RMSD values around 0.15–0.20 nm, while the ligand (black) showed expected flexibility with RMSD fluctuations between 0.05–0.25 nm. Notably, we found that the complete protein–ligand complex (red) exhibited remarkable stability with RMSD values consistently around 0.12–0.15 nm.

To investigate local flexibility, we calculated the RMSF profile across all 8000 atoms (Figure 5B). Our analysis revealed varying degrees of atomic mobility throughout the structure, with most regions displaying fluctuations between 0.1–0.2 nm. We identified several distinct peaks reaching up to 0.4–0.5 nm, indicating localized areas of higher flexibility.

We then examined the hydrogen bonding patterns between raloxifene and RdRP (Figure 5C) to understand their binding dynamics. Our results showed that the number of hydrogen bonds fluctuated primarily between 1–3, with occasional peaks reaching 5 bonds, particularly evident between 10–15 ns. We interpreted this pattern as indicating a stable yet flexible binding interaction maintained throughout the simulation.

To further characterize the binding stability, we measured the average distance between the protein and ligand centers of mass (Figure 5D). We observed the simulation period, starting at approximately 1.0 nm and reaching about 1.2 nm by 30 ns. Despite this minor shift, we confirmed that the overall binding remained stable, as supported by the consistent hydrogen bonding patterns.

To validate simulation quality, we monitored system energetics. We found that the total energy (Figure 5E) remained stable around −5.48 × 10⁵ kJ/mol throughout the simulation, confirming proper energy conservation. Additionally, we maintained temperature control (Figure 5F) effectively at the target temperature of 300 K, with fluctuations typically within ±5 K, verifying appropriate thermal regulation during the simulation period.

## 4. Discussions

Our multi-model machine learning framework presents a novel approach for identifying potential RNA polymerase inhibitors from FDA-approved drugs. Through comprehensive analyses, we have demonstrated the effectiveness of combining traditional machine learning algorithms with deep learning methods to enhance predictive accuracy and reliability.

The five traditional machine learning classifiers we evaluated (ExtraTreesClassifier, RandomForestClassifier, LGBMClassifier, BernoulliNB, and BaggingClassifier) demonstrated robust performance, with accuracy and ROC-AUC values ranging from 0.70 to 0.75. Notably, ensemble learning approaches, particularly tree-based methods, emerged as the most effective for identifying potential RNA polymerase inhibitors. This finding aligns with previous studies that have highlighted the superiority of ensemble methods for chemical structure-based activity prediction.

Our CNN deep learning model, while exhibiting slightly lower performance metrics compared to traditional classifiers, provided complementary predictive value to the overall framework. The model’s ability to process complex molecular representations and maintain high specificity (0.77) when tested with external validation data underscores its utility in virtual screening applications. The Venn diagram analysis further validates our multi-model approach, revealing that 221 compounds were correctly identified by all six models, establishing a high-confidence consensus set.

Beyond our primary screening approach, we recognized that many compounds in the FDA-approved drug library from Selleck had been overlooked for various reasons, including discontinued usage, limited market presence, or safety concerns that emerged post-approval. To address this limitation and focus on drugs with established safety profiles and current clinical relevance, we implemented a secondary filtration step using RxNav, a specialized tool provided by the National Library of Medicine. This additional screening process enabled us to refine our analysis to include only FDA-approved drugs currently available on the market with well-documented safety records, thereby enhancing the practical applicability, safety assurance, and translational potential of our findings (Figure 4A).

The molecular docking studies further strengthened our findings, identifying raloxifene as a promising RNA polymerase inhibitor candidate. The similarities between raloxifene’s binding pose and the positive control, coupled with its favorable binding energy (−8.8 kcal/mol), suggest potential inhibitory activity comparable to known inhibitors.

Despite irbesartan’s binding pose differing from both our positive control and raloxifene, this alternative binding mode could still meaningfully affect RNA polymerase activity. First, the high binding affinity indicates thermodynamically favorable binding that could effectively compete with natural substrates or disrupt essential protein dynamics. Second, although positioned differently, irbesartan could interact with several key residues critical for polymerase function. These interactions could potentially interfere with template positioning, nucleotide recognition, or the conformational changes required during the catalytic cycle. Third, while some inhibitors directly compete with incoming nucleotides or the RNA template, others function through allosteric mechanisms by inducing conformational changes that impair catalysis. Irbesartan could potentially employ such allosteric mechanisms to disrupt polymerase function. The inhibitory effect of irbesartan on RNA polymerase and its mechanism warrants further investigation.

Molecular dynamics simulations reveal that both raloxifene (Figure 5) and the positive control compound (Figure S1) maintain stable interactions with RdRP despite exhibiting distinct binding profiles. The RMSD trajectories demonstrate that raloxifene forms a more structurally rigid complex (RMSD ~0.13 nm) compared to the positive control (~0.2 nm), though both remained consistently bound throughout the simulation period. Hydrogen bond analysis shows the positive control maintained a stronger network (2–6 hydrogen bonds) compared to raloxifene’s more variable pattern (0–5 hydrogen bonds). The energetic analysis further differentiates the binding modes, with the positive control exhibiting a more favorable total energy (−7.4 × 10^5^ KJ/mol versus −5.47 × 10^5^ KJ/mol for raloxifene). Interestingly, despite these differences in interaction patterns, both compounds target overlapping binding regions within RdRP, as evidenced by similar RMS fluctuation profiles across the protein structure. Temperature stability (maintained at ~300 K) and consistent average distance measurements throughout the simulations further support that both ligands achieve high binding stability within the same critical pocket of RdRP, suggesting this region can accommodate different chemical scaffolds while maintaining functionally relevant interactions.

The structural comparison of RdRPs from various viruses, including picornaviruses (PDB ID: 1RA6, green), noroviruses (PDB ID: 4NRT, orange), hepatitis C virus (PDB ID: 1QUV, blue), and SARS-CoV-2 (PDB ID: 7L1F, pink), demonstrates significant structural conservation in their catalytic domains (Figure S2). Figure S2A shows the overall superposition of these viral polymerases, while Figure S2B highlights a zoomed-in view of the potential binding pocket. Figure S2C provides a sequence alignment visualization that correlates with the structural similarities. The three-dimensional architecture of the active site and surrounding regions shows remarkable conservation across these evolutionarily distant viral families. The binding pocket highlighted in Figure S2B,C reveals conservation of key structural elements that could accommodate raloxifene binding, including similar secondary structure elements and spatial arrangement. This structural conservation suggests the potential broad-spectrum antiviral activity of raloxifene against RNA viruses from different families.

The repurposing of FDA-approved drugs offers significant advantages in drug development, including reduced development timelines and well-established safety profiles [24]. Our identification of raloxifene, currently approved for osteoporosis treatment [25] and breast cancer prevention [26], aligns with recent research demonstrating its efficacy against various viral infections. Raloxifene has shown promising results in treating Ebola, influenza A, and hepatitis C viruses, while also displaying potential for repurposing against SARS-CoV-2 infection [27,28,29]. Notably, RdRP plays a crucial role in the proliferation of SARS-CoV-2 [30], hepatitis C [31], influenza A [32], and Ebola [33] viruses. Our computational findings suggesting that raloxifene can inhibit RdRP activity provide a mechanistic explanation for its previously observed broad antiviral properties, indicating that raloxifene may prevent viral proliferation through RdRP inhibition.

While our computational framework provides compelling evidence for raloxifene’s potential as an RNA polymerase inhibitor, experimental validation studies, including in vitro enzymatic assays and cellular antiviral activity assessments, are essential next steps. Additionally, structure–activity relationship studies could further optimize raloxifene or guide the development of derivative compounds with enhanced potency and selectivity.

## 5. Conclusions

Our multi-model machine learning framework represents a valuable approach for identifying novel RNA polymerase inhibitors from FDA-approved drugs. The complementary nature of traditional machine learning and deep learning models, coupled with molecular docking and dynamics simulations, provides a comprehensive computational pipeline for drug repurposing efforts targeting viral RNA polymerases. The identification of raloxifene as a promising candidate highlights the potential of repurposing drugs against RNA virus infections.

## Data Availability

Data will be made available on request.

## Supplementary Materials

The following supporting information can be downloaded at: https://www.mdpi.com/article/10.3390/cimb47050315/s1.
