# Supplementary material for: A Multi-Model Machine Learning Framework for Identifying Raloxifene as a Novel RNA Polymerase Inhibitor from FDA-Approved Drugs
Source: Curr Issues Mol Biol. 2025 Apr 28;47(5):315. doi: 10.3390/cimb47050315 (PMC12110393; doi:10.3390/cimb47050315)
Supplement: Supplementary file 1 [file cimb-47-00315-s001.zip › cimb-3573298-supplementary.pdf]

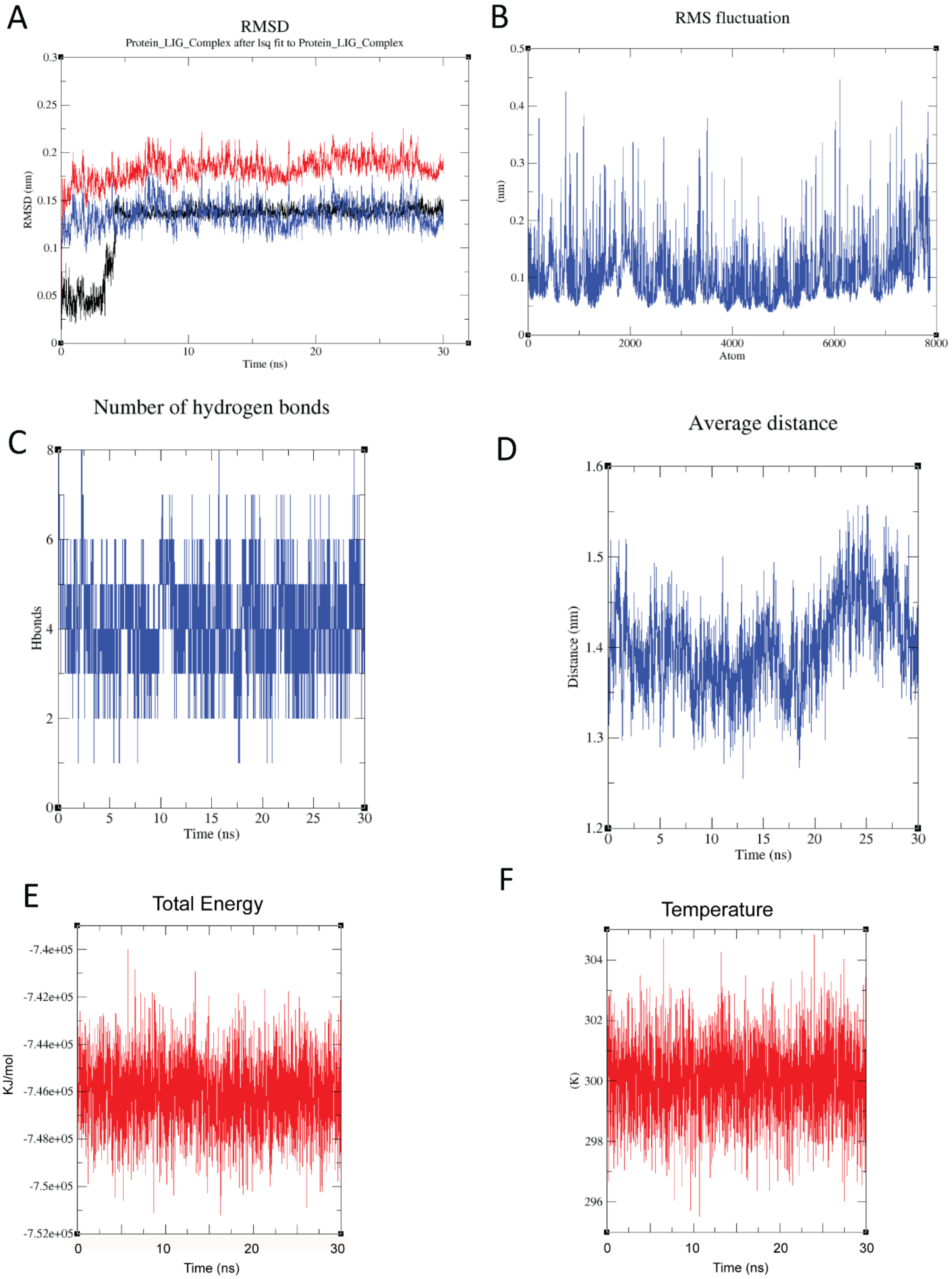
A
RMSD
Protein_LIG_Complex after lsq fit to Protein_LIG_Complex
RMSD (nm)
Time (ns)
B
RMS fluctuation
(nm)
Atom
C
Number of hydrogen bonds
Hbonds
Time (ns)
D
Average distance
Distance (nm)
Time (ns)
E
Total Energy
KJ/mol
Time (ns)
F
Temperature
(K)
Time (ns)

Figure S1. Molecular dynamics simulation analysis of RdRP (protein)-Positive Control (ligand) complex stability and interactions over 30 nanoseconds (ns). (A) Root mean square deviation (RMSD) analysis demonstrating the conformational evolution of the ligand (black), protein backbone (blue), and complete protein-ligand complex (red). Values were calculated after least-squares fitting to the initial structure. (B) Root mean square fluctuation (RMSF) per atom, calculated over 8000 atoms. Values represent atomic positional fluctuations from their time-averaged positions. (C) Time-dependent analysis of intermolecular hydrogen bonds between the protein and ligand, measured as the total number of hydrogen bonds present at each time point. (D) Average distance evolution between protein and ligand centers of mass throughout the simulation, measured in nanometers (nm). (E) Total energy profile of the system, including potential and kinetic energy contributions, measured in kJ/mol. (F) Temperature regulation profile maintaining the system at approximately 300 K. Temperature is measured in Kelvin (K).

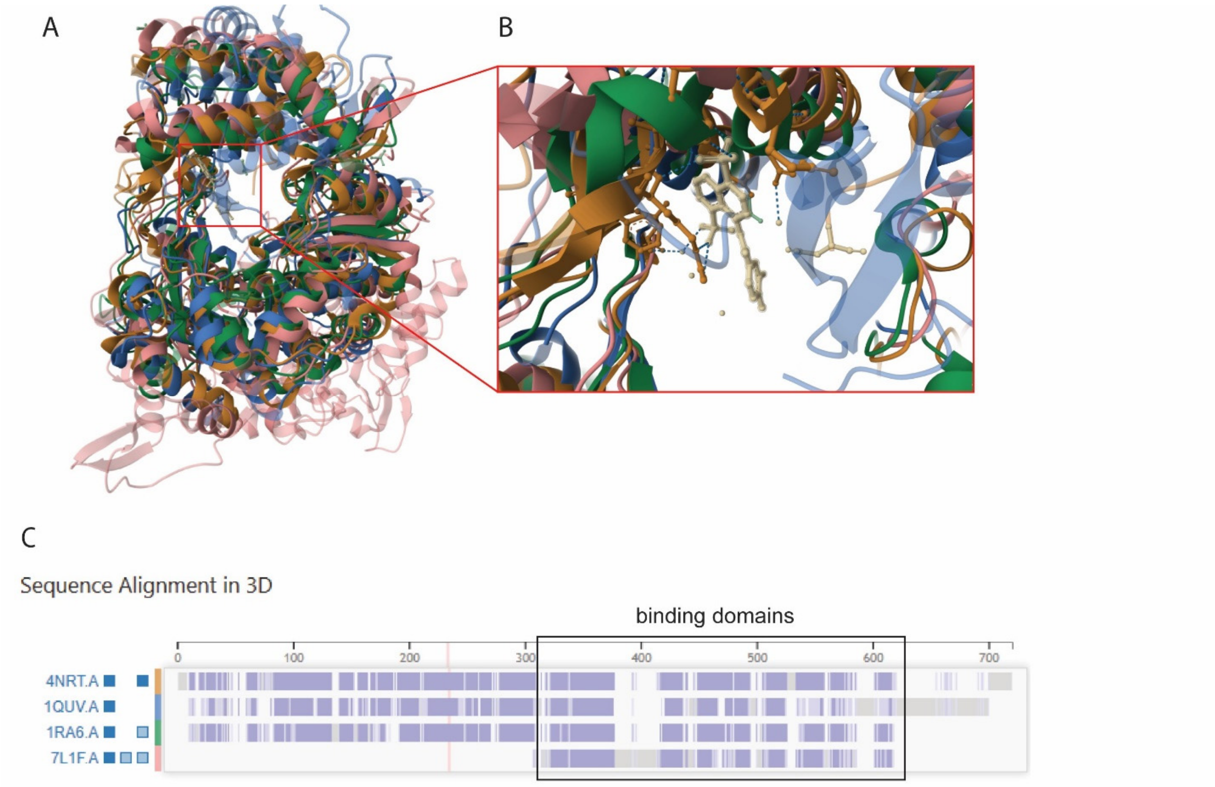

Figure S2. Structural comparison of RNA-dependent RNA polymerases from various viruses

(A) Superposition of RdRp structures from picornavirus (1RA6, green), norovirus (4NRT, orange), hepatitis C virus (1QUV, blue), and SARS-CoV-2 (7L1F, pink). The overall tertiary structure shows the conserved right-hand architecture characteristic of viral polymerases.

(B) Magnified view of the potential binding pocket (red box in panel A), highlighting the structural conservation in the active site region across all four viral polymerases. The spatial arrangement of key structural elements suggests a common binding mechanism for small-molecule inhibitors like Raloxifene.

(C) Sequence alignment in 3D showing the conservation patterns across the four viral RdRps. Purple bars indicate regions of sequence similarity, with darker shades representing higher conservation. The scale bar indicates amino acid positions from 0 to 700.
